# SEMA6A/RhoA/YAP axis mediates tumor-stroma interactions and prevents response to dual BRAF/MEK inhibition in BRAF-mutant melanoma

**DOI:** 10.1186/s13046-022-02354-w

**Published:** 2022-04-19

**Authors:** Rossella Loria, Valentina Laquintana, Stefano Scalera, Rocco Fraioli, Valentina Caprara, Italia Falcone, Chiara Bazzichetto, Marta Di Martile, Laura Rosanò, Donatella Del Bufalo, Gianluca Bossi, Isabella Sperduti, Irene Terrenato, Paolo Visca, Silvia Soddu, Michele Milella, Gennaro Ciliberto, Rita Falcioni, Virginia Ferraresi, Giulia Bon

**Affiliations:** 1 Cellular Network and Molecular Therapeutic Target Unit, IRCCS Regina Elena National Cancer Institute, Via Elio Chianesi 53, 00144 Rome, Italy; 2 Department of Pathology, IRCCS Regina Elena National Cancer Institute, Rome, Italy; 3 SAFU Laboratory, Department of Research, Advanced Diagnostic, and Technological Innovation, IRCCS Regina Elena National Cancer Institute, Rome, Italy; 4 Oncogenomic and Epigenetic Unit, IRCCS Regina Elena National Cancer Institute, Rome, Italy; 5 Preclinical Models and New Therapeutic Agents Unit, IRCCS Regina Elena National Cancer Institute, Rome, Italy; 6 Division of Medical Oncology 1, IRCCS Regina Elena National Cancer Institute, Rome, Italy; 7 Institute of Molecular Biology and Pathology, National Research Council (CNR), Rome, Italy; 8 Biostatistics and Bioinformatic Unit, Scientific Direction, IRCCS Regina Elena National Cancer Institute, Rome, Italy; 9 Section of Oncology, Department of Medicine, University of Verona and Verona University Hospital Trust (AOUI Verona), Verona, Italy; 10 Scientific Directorate, IRCCS Regina Elena National Cancer Institute, Rome, Italy

**Keywords:** Semaphorin SEMA6A, Melanoma, Dual BRAF/MEK inhibition, Actin cytoskeleton remodeling, YAP, Tumor microenvironment

## Abstract

**Background:**

Despite the promise of dual BRAF/MEK inhibition as a therapy for BRAF-mutant (BRAF-mut) melanoma, heterogeneous responses have been observed in patients, thus predictors of benefit from therapy are needed. We have previously identified semaphorin 6A (SEMA6A) as a BRAF-mut-associated protein involved in actin cytoskeleton remodeling. The purpose of the present study is to dissect the role of SEMA6A in the biology of BRAF-mut melanoma, and to explore its predictive potential towards dual BRAF/MEK inhibition.

**Methods:**

SEMA6A expression was assessed by immunohistochemistry in melanoma cohort RECI1 (*N* = 112) and its prognostic potential was investigated in BRAF-mut melanoma patients from DFCI and TCGA datasets (*N* = 258). The molecular mechanisms regulated by SEMA6A to sustain tumor aggressiveness and targeted therapy resistance were investigated in vitro by using BRAF-mut and BRAF-wt melanoma cell lines, an inducible SEMA6A silencing cell model and a microenvironment-mimicking fibroblasts-coculturing model. Finally, SEMA6A prediction of benefit from dual BRAF/MEK inhibition was investigated in melanoma cohort RECI2 (*N* = 14).

**Results:**

Our results indicate higher protein expression of SEMA6A in BRAF-mut compared with BRAF-wt melanoma patients and show that SEMA6A is a prognostic indicator in BRAF-mut melanoma from TCGA and DFCI patients cohorts. In BRAF-mut melanoma cells, SEMA6A coordinates actin cytoskeleton remodeling by the RhoA-dependent activation of YAP and dual BRAF/MEK inhibition by dabrafenib+trametinib induces SEMA6A/RhoA/YAP axis. In microenvironment-mimicking co-culture condition, fibroblasts confer to melanoma cells a proliferative stimulus and protect them from targeted therapies, whereas SEMA6A depletion rescues the efficacy of dual BRAF/MEK inhibition. Finally, in BRAF-mut melanoma patients treated with dabrafenib+trametinib, high SEMA6A predicts shorter recurrence-free interval.

**Conclusions:**

Overall, our results indicate that SEMA6A contributes to microenvironment-coordinated evasion of melanoma cells from dual BRAF/MEK inhibition and it might be a good candidate predictor of short-term benefit from dual BRAF/MEK inhibition.

**Supplementary Information:**

The online version contains supplementary material available at 10.1186/s13046-022-02354-w.

## Background

Melanoma is characterized by a high frequency of genetic [1] and epigenetic [2–4] deregulations compared to other tumor types, with the majority of mutations affecting the mitogen-activated protein kinase (MAPK) pathway [5].

BRAF mutation is the most common MAPK pathway aberration, occurring in 40-60% of melanoma cases. The introduction of BRAF-targeted therapies [6] and, more recently, of combination therapy with BRAF and MEK inhibitors, such as dabrafenib+trametinib [7–9], has resulted in dramatic improvements in terms of overall survival (OS) and progression-free survival (PFS) in patients affected by BRAF-mutant (BRAF-mut) advanced melanoma. Moreover, immune checkpoint inhibitors (anti-CTLA4 and anti-PD1) are an additional promising therapeutic option available that further improved outcomes in patients with metastatic melanoma [10–13]. However, some patients benefit from dual BRAF/MEK inhibition much more than others [9, 14, 15], indicating that the identification of novel predictive biomarkers is needed to optimize individualized treatment strategies.

By an unbiased screening of different single cell clones derived from the same melanoma patient and mutually carrying BRAF^V600E^ or NRAS^Q61R^ mutations, we previously identified semaphorin SEMA6A as a protein whose expression is associated with BRAF^V600E^ mutation. In addition, we observed that SEMA6A regulates actin cytoskeleton remodeling, thereby sustaining proliferation and survival of BRAF-mut melanoma cells [16]. Later on, SEMA6A deregulation in BRAF^V600E^ as compared with BRAF wt melanoma patients has been reported by others [17].

Semaphorins belong to a family including more than 20 members; some of them have been shown to modulate angiogenesis, invasiveness, and metastatization by regulating monomeric GTPases, cell-substrate adhesion, and cytoskeletal dynamics [18–20]. More recently, the involvement of semaphorins in the complex signal exchange between tumor and its microenvironment is emerged and supported by a large body of evidence [21–29]. Of relevance, tumor microenvironment plays an important role in protecting cancer cells from the anti-tumor activity of BRAF and MEK inhibitors through paradoxic upregulation of signaling pathways and survival factors [30–33]. A very first response of cancer cells to stimuli from tumor microenvironment is represented by actin cytoskeleton remodeling to promote survival, migration, and metastatization [34, 35]. A critical role in this process is played by the Rho family of small GTPases that, through the activation of downstream targets including YAP, coordinates stress fibers and actin bundles formation [36].

YAP is a coactivator of transcription factors and a Hippo suppressor pathway transducer with tumorigenic potential in mouse model [37], and pro-invasive properties in melanoma [38]. Nuclear accumulation of YAP has been described in a variety of cancers [39–43], resulting in activation of target genes mainly involved in cell proliferation. Remarkably, YAP activation has been reported to be associated with stemness [44], conversion of normal fibroblasts to cancer-associated fibroblasts (CAFs) [45], and resistance to BRAF and/or MEK inhibitors [46, 47]. Specifically, YAP confers resistance to BRAF inhibitors by inducing actin cytoskeleton remodeling in melanoma [48] and other tumors [49], and cytoskeletal tension itself has been shown to affect chemotherapeutic drug sensitivity of cancer cells [50].

In the present study, we observed that SEMA6A expression is higher in BRAF-mut than in BRAF-wt melanoma patients and has a prognostic significance in BRAF-mut patients from TCGA and DFCI large cohorts. Mechanistically, we show that in BRAF-mut melanoma cells, SEMA6A remodels actin cytoskeleton by activating the RhoA-YAP axis. Furthermore, the activity of SEMA6A/RhoA/YAP axis is induced by dual BRAF/MEK inhibition by dabrafenib+trametinib and, in an environmental-mimicking condition of melanoma cells and fibroblasts co-culture, is associated with reduced targeted therapy efficacy. These results were confirmed in BRAF-mut melanoma patients treated with dabrafenib+trametinib, where high SEMA6A expression predicts shorter progression-free survival upon therapy. Overall these data support the involvement of SEMA6A in fibroblasts-induced protection of melanoma cells from the anti-tumor activity of dual BRAF/MEK inhibition and indicate that SEMA6A might be a good candidate predictor of short-term benefit from dual BRAF/MEK blockade.

## Methods

### Patient cohorts

For SEMA6A protein expression two patient cohorts were used; the RECI1 cohort, including 59 BRAF^V600E^ and 53 BRAF-wt melanoma patients treated at the Regina Elena National Cancer Institute, was used to compare SEMA6A expression in BRAF-mut versus BRAF-wt tumors. Bioptic specimens were collected from the metastatic lesions of involved patients. The RECI2 cohort was used to assess the predictive role of SEMA6A expression in 14 BRAF-mut melanoma patients admitted to dabrafenib+trametinib at Regina Elena National Cancer Institute. Based on recurrence-free interval, RECI2 patients were categorized as short-term and long-term responders; a cut-off value of 12 months was established. Information on clinical and histopathological features, anti-tumoral therapies and related outcomes were retrieved from patients’ medical records by specifically trained research assistants; written informed consent from all patients was obtained in accordance with the Declaration of Helsinki. TCGA (DOI: 10.1016/j.cell.2018.03.022) and DFCI (DOI: 10.1038/s41591-019-0654-5) melanoma cohorts were used to investigate the prognostic role of SEMA6A in BRAF-mut melanoma. Melanoma cases were referred to as “early” when stage was 0, I, and II, and “advanced” when stage was III and IV. Genomic and clinical data from the two cohorts were downloaded from cBioportal. Only patients with BRAF mutation were considered for the analyses. RNA-seq data were obtained from Firebrowse (http://firebrowse.org/). SEMA6A expression from RNA-seq data was quantified through TPM (Transcripts per Million) normalization. Patients were classified as SEMA6A High and Low expression by calculating the relative TPM value for each sample in the patient cohort (*N* = 20).

### Immunohistochemistry

Formalin-fixed paraffin-embedded sections were analyzed as described [41]. Antigen retrieval was performed at 96 °C (10 mM/L citrate buffer, pH 6) for 20 min. Sections from specimens of RECI1 and RECI2 cohorts were incubated with the primary antibody anti-SEMA6A 1:50 (HPA031265; SIGMA-Aldrich, St. Luis, MO USA) for 30 min at room temperature. Immunoreactions were revealed by Bond Polymer Refine Detection Kit according to manufacturer’s procedure (Leica Biosystems) in an automated autostainer Bond III Leica Biosystems. Diaminobenzidine was used as chromogenic substrate. Microscope Nikon ECLIPSE 55i with digital camera HESP Technology was used. Scale bars 30 μm. The study was reviewed and approved by the ethical committee of Regina Elena National Cancer Institute, and informed consent was obtained from all patients. For IHC, the secondary antibody was used as internal control.

### Cell lines, co-culture, and treatments

The Human Fibroblast BJ were obtained from the American Type Culture Collection (ATCC) and maintained in DMEM medium supplemented with 10% FBS and 1% penicillin/streptomycin (Gibco, Life Technologies, Milan, Italy). BRAF-wt/NRAS-wt ME1007 and BRAF-wt/NRAS-mut ME4405, derived from lymph node metastases, and BRAF-wt/NRAS-mut 2/17 and BRAF^V600E^/NRAS-wt 2/59, derived from subcutaneous metastasis, were isolated from surgical specimens of melanoma patients, not previously subjected to chemotherapy and admitted to Fondazione IRCCS Istituto Nazionale dei Tumori, Milan (Sensi M et al., 2006; Daniotti M et al., 2004). M14 and C32 cell lines were obtained from the American Type Culture Collection (ATCC).

To obtain an inducible SEMA6A depletion, 2/59 cells were transduced using shERWOOD UltramiR lentiviral inducible shRNA (pZIP-TRE3G) according to the manufacturer’s guidelines (Transomic Technologies, Huntsville, AL USA). Stable cells were generated by antibiotic selection with 1 μg/mL puromycin for 14 days. We obtained a control cell line by using a non-specific shRNA (2/59 shCtrl) and two cell lines inducible for SEMA6A depletion by using two specific shRNAs (2/59 shSEMA6A A3 and H2). To induce shRNAs and GFP reporter gene expression, cells were treated with doxycycline at 0,5 μg/mL. All melanoma cell lines were maintained in RPMI medium supplemented with 10% FBS and 1% penicillin/streptomycin (Gibco, Life technologies, Milan, Italy). All cell lines were periodically tested for mycoplasma contamination.

For co-cultures, BJ were plated and 3 days after 2/59 shCtrl, A3 and H2 were added to BJ monolayer cultures without discard the culture medium.

For treatments in vitro, trametinib and dabrafenib (Selleckchem, Houston, TX, USA) were used at 5 nM and 0,1 μM concentrations respectively. The drugs were stored as stock solutions at − 20 °C and diluted just before use.

### Antibodies

Anti-Sema6A (ab72369) was from Abcam (Cambridge, UK), anti-Lamin A (#86846), anti-Akt (#9272), anti-phospho-ser473-Akt (#9271), anti-Erk1/2 (#9102), anti-phospho-Thr202/Tyr204-Erk 1/2 (#9101), anti-YAP (#12395), anti-phospho-ser127-YAP (#13008), anti-p65 (#8242), anti-phospho-ser536-p65 (#3033), anti-Tubulin (#2125) were from Cell Signaling (Danvers, MA USA), anti-Hsp-70 (ab-83,392) and anti-GAPDH (ab-81,594) were from Immunological Sciences (Rome, Italy). HRP-conjugated secondary antibodies were from Bio-Rad (Hercules, CA USA).

### Western blotting

All cell lines, before and after treatment, were lysed using RIPA buffer, analyzed by SDS-PAGE and probed (WB) with antibodies of interest and secondary HRP-conjugated antibodies. Signals were detected by Amersham ECL Prime Western Blotting Detection Reagent (GE Healthcare Life Sciences, Chicago, IL USA) and by LuminataTM Classico Western HRP substrate (Millipore, Burlington, MA USA). Same amount of total protein from three independent experiments were pooled and analyzed. Each experiment was repeated at least three times.

### Nucleic and cytoplasmic fractionation

Nucleic and cytoplasmic fractions, derived from 2/59 shCtrl and shSEMA6A A3 and H2 cells 72 h post-induction, untreated or treated with RhoA activator I, were obtained using NE-PER Nuclear and Cytoplasmic Extraction Reagents (#78835; Thermo Scientific, Rockford IL USA) according to the manufacturer’s guidelines. Each experiment was repeated at least three times.

### RhoA activation assay and immunofluorescence analysis

2/59 shCtrl and shSEMA6A A3 and H2 cells were plated on 100 mm dishes or poly-l lysine coated slides 24 h post-induction. After 48 h cells were treated with 1 U/mL RhoA activator I, according to the manufacturer’s guidelines (#CN01; Cytoskeleton, Denver, CO USA). The levels of RhoA activation in untreated or treated cells were analyzed by RhoA activation assay (#BK036; Cytoskeleton, Denver CO USA) performed according to the manufacturer’s guidelines.

For immunofluorescence experiments, cells plated at a subconfluent state, untreated or treated with RhoA activator I as described above, were stained with anti-YAP (sc-376,830; Santa Cruz, Dallas TX, USA) or Alexa Fluor 555 Phalloidin (#8953; Cell Signaling, Danvers, USA) and counterstained with Hoechst to highlight nuclei (SIGMA-Aldrich, St. Luis, MO USA). 2/59 and ME4405 cells plated on poly-l lysine coated slides were treated with trametinib, dabrafenib and their combination, and stained as above. The quantification of YAP localization and stress fiber positive cells was performed by inspecting at least five fields/slide using a 60X magnification in three independent experiments. The results were reported as percentage of whole number of counted cells.

Microscope OLYMPUS BX53 was used to evaluate fluorescence. Scale bars 10 μm. Each assay was carried out in triplicate and repeated at least three times.

### Crystal violet assay

Following doxycycline induction for 24 h, 2/59 shCtrl and shSEMA6A A3 and H2 cells plated on 24-well plates were treated with trametinib, dabrafenib and/or their combination at specified concentrations. Seventy-two hours later the cells were fixed with 4% formaldehyde for 15 min and stained with 0.1% crystal violet solution in 10% ethanol for 40 min. At the end of incubation the medium was removed, cells were allowed to dry at room temperature and dissolved in isopropanol, and optical density was measured at 570 nm using an ELISA plate reader. Each assay was carried out in quadruplicate and repeated at least three times.

### Cell sorting and IncuCyte® analyses

BJ were plated on 12-well plates or 60 mm dishes and 3 days later shCtrl, A3 and A H2 2/59, following a 72 h-induction, were added to BJ monolayer cultures without discard the culture medium. The day after cells were treated with trametinib, dabrafenib or their combination.

After 48 h melanoma cells seeded on 60 mm dishes were separated from the fibroblasts by BD FACSMelody Cell Sorter, gating on the GFP signal and collecting only clearly GFP positive melanoma cells. The purity of the FACS-separated melanoma fractions (i.e. no contamination with fibroblasts) is guaranteed since the gating was stringent. Total cell lysate extracted from sorted melanoma cells were analyzed by western blot.

Co-culture performed on 12-well plates were incubated in IncuCyte® and monitored non-invasively by a planned acquisition of images every 12 h for 156-180 h. Images were analyzed by software supplied by Sartorius. Each experiment was carried out in triplicate and repeated at least three times.

### Statistical analyses

Data were reported as the mean of the three replicates ± standard deviation (SD). Each experiment was performed independently. To compare continuous variables two-tailed Student’s t-tests with Benjamini–Hochberg correction was performed while Pearson’s Chi-square test was used to compare categorical variables. One-way ANOVA with Tukey HSD test was used for multiple sample comparison. Survival analysis was conducted through the use of Kaplan-Meier method and the Log-rank test was used to individuate potential differences between subgroups. *P*-value < 0.05 was considered significant. Statistical analyses were performed with SPSS software (SPSS version 21.0, SPSS Inc., Chicago, IL) and in-house scripting in R environment.

## Results

### SEMA6A is highly expressed and has prognostic relevance in BRAF-mut melanoma patients

**Fig. 1:**
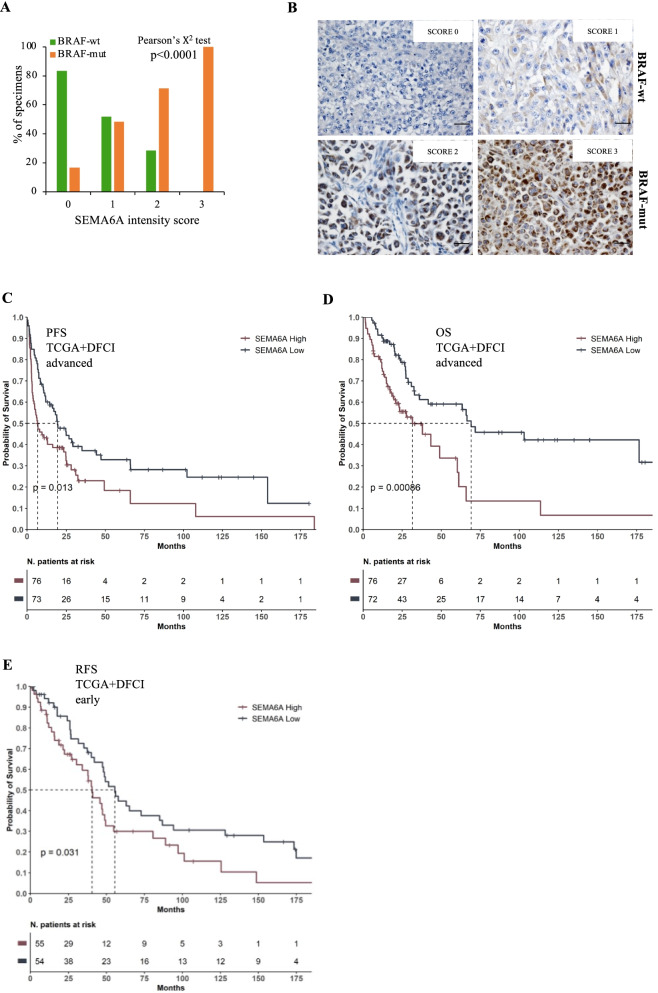
SEMA6A is a prognostic indicator in BRAF-mut melanoma patients. **A**: SEMA6A Immunohistochemical assessment in BRAF-wt and BRAF-mut melanoma specimens of RECI1 cohort. Pearson’s Chi-square test revealed a significant association between SEMA6A expression and BRAF mutation (*p* < 0,0001). **B**: Representative sections from 2 BRAF-wt and 2 BRAF-mut melanoma patients, stained with anti-SEMA6A antibody. **C**: Progression-free Survival (PFS) and **D**: Overall Survival (OS) in patients with with high and low SEMA6A (BRAF-mut advanced melanoma). **E**: Relapse-free survival (RFS) in patients with high and low SEMA6A (BRAF-mut early melanoma) from the cohorts TCGA and DFCI

We have previously shown preferential expression of SEMA6A in BRAF-mut cell lines and melanoma lymph-node metastases compared with BRAF-wt ones [16]. We first verified the association of SEMA6A protein expression with BRAF mutation in the RECI1 cohort, which includes 112 metastatic advanced melanoma patients surgically treated at the Regina Elena National Cancer Institute. SEMA6A protein expression was measured by immunohistochemistry (IHC) and assigned an intensity score (IS) value ranging from 0 to 3. We then dichotomized SEMA6A into two categories: “*low*” if IS was 0 or 1 and “*high*” if score was 2 or 3. Table 1 shows the main patient- and tumor-related characteristics. Briefly, 59 patients (52,7%) were affected by BRAF-mut disease and 53 (47,3%) were BRAF-wt. SEMA6A was high in 36 (32,1%) and low in 76 (67,9%) patients. As shown in Fig. 1A and in representative IHC panels of Fig. 1B, we found a statistically significant association between high SEMA6A and BRAF mutation (Pearson’s Chi-square test *p* < 0,0001) (Fig. 1A). Notably, 100% of samples with SEMA6A IS 3 were BRAF-mut. In addition, we performed SEMA6A IHC in subsequent metastatic lesions at diagnosis and at progression in three patients. As shown in Table 2, SEMA6A levels progressively increased, suggesting that SEMA6A may have prognostic relevance in BRAF-mut melanoma. To verify this hypothesis, we analyzed mRNA data from TCGA and DFCI large datasets; advanced and early melanoma patients were analyzed separately. When focusing on patients with advanced melanoma (*n* = 149), patients with high SEMA6A expression had reduced PFS and OS than those with low SEMA6A (PFS: 6.76 months vs. 19.50 months; Log-Rank *p* = 0.013; OS: 31.6 months vs. 69.1 months; Log-Rank 0.0008) (Fig. 1C and D). In patients with early melanoma (*n* = 109), high SEMA6A was significantly associated with reduced relapse-free survival (RFS) (40.5 months vs. 55.5 months; Log-Rank *p* = 0.031) (Fig. 1E). Overall these data support the involvement of SEMA6A in the biology of BRAF-mut melanoma and indicate that SEMA6A is a prognostic indicator in this subset of patients.

**Table 1:** Clinicopathological characteristics of metastatic advanced melanoma patients from the RECI1 cohort (*N* = 112)

Characteristics	*N* (%)
**Age yr**, median (range)	57 (25-80)
**Gender**	
*Male*	55 (49.1)
*Female*	57 (50.9)
**BRAF status**	
*mut*	59 (52.7)
*wt*	53 (47.3)
**SEMA6A**	
*Low*	76 (67,9)
*High*	36 (32,1)
**Metastatic Site**	
*Lymph Node*	55 (49.1)
*Cutaneous/subcutaneous*	30 (26.8)
*Lung*	14 (12.5)
*Brain*	6 (5.4)
*Liver*	3 (2.7)
*Other*	4 (3.6)

**Table 2:** Progressively increasing expression of SEMA6A in subsequent metastatic lesions from three patients

Patient	Diagnosis		Progression	
	% of SEMA6A+ cells	Tumor site	% of SEMA6A+ cells	Tumor site
1	60%	Back	70%	Back
2	30%	Lung	80%	Lung
3	10%	Leg	70%	Occipital fragment

### SEMA6A regulates actin cytoskeleton remodeling by activating the RhoA/YAP axis in BRAF-mut melanoma cells

**Fig. 2:**
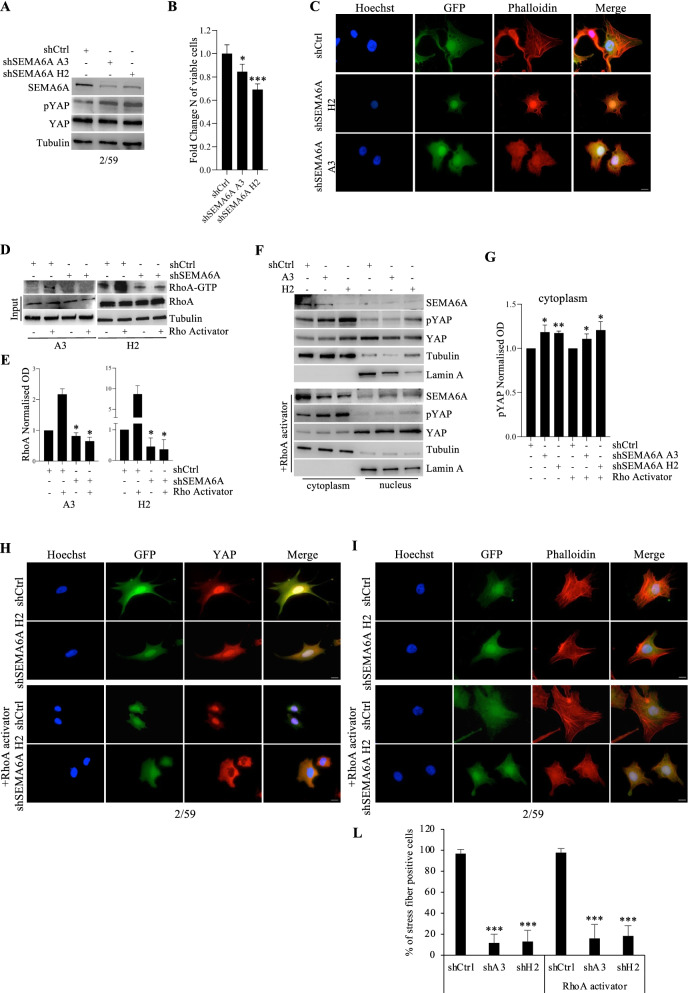
SEMA6A depletion reduces RhoA activity and induces YAP phosphorylation and cytoplasmic retention in BRAF-mut melanoma cells. **A**: western blot (WB) analysis of SEMA6A, phosphorylated and total YAP was performed on total cell extracts from inducible shCtrl and shSEMA6A A3 and H2 2/59 cells upon silencing induction. The anti-tubulin antibody was used to validate equivalent amount of loaded proteins in each lane. **B**: Fold change number of viable A3 and H2 cells compared with shCtrl cells 72 h post-induction. The results are presented as mean +/− standard deviation of three independent experiments (*p < 0.05; *** *p* < 0,0001). **C**: shCtr and SEMA6A-depleted A3 and H2 cells were plated on poly-l lysine coated slides and stained with Phalloidin (red signal). GFP reporter gene expression revealed successful silencing induction. The cells were counterstained with Hoechst to highlight nuclei. Scale bar 10 μm. **D**: WB analysis of activated RhoA (RhoA-GTP) pulled down from cell lysates and total RhoA on cell extracts from shCtrl and SEMA6A-depleted 2/59 cells, treated or not with 1 U/mL RhoA activator. The anti-tubulin antibody was used to validate equivalent amount of loaded proteins in each lane. **E**: Densitometric analysis of RhoA-GTP normalized to RhoA obtained from shCtrl and SEMA6A-depleted A3 and H2 cell populations treated or not with 1 U/mL RhoA activator. The results are presented as mean+/− standard deviation of three independent experiments (*p < 0.05). **F**: Cytoplasmic and nuclear fractions extracted from shCtr and A3 and H2 cells, treated or not with RhoA activator, were analyzed by WB for the expression of SEMA6A, phosphorylated and total YAP. Lamin A and α-tubulin were used to validate purity of nuclear and cytoplasmic extracts respectively. **G**: Densitometric analysis of p-YAP normalized to total YAP in cytoplasmic fraction of shCtrl, A3 and H2 cells treated or not with 1 U/mL RhoA activator. The results are presented as mean+/− standard deviation of three independent experiments (*p < 0.05; ** *p* < 0,001).. **H**: shCtr and SEMA6A-depleted H2 cells plated on poly-l lysine coated slides were treated or not with RhoA activator and stained with anti-YAP (red signal) or **I**: with Phalloidin (red signal). Scale bar 10 μm. **L:** the number of stress fibers containing cells from the experiment shown in panel I is reported as percentage and at least 200 cells were counted per experiment. The results are presented as mean+/− standard deviation of three independent experiments (*** *p* < 0,0001)

We have previously identified SEMA6A as a BRAF-mut-associated protein that might contribute to actin cytoskeleton remodeling [16]. Our previous findings indicate that the depletion of SEMA6A by siRNA has dramatic effect in terms of cell death; thus, to unravel the molecular mechanism by which SEMA6A regulates actin stress fibers dynamics, we aimed to exploit a cell model that would allow us to modulate SEMA6A expression and avoid massive cell death. For this purpose, the BRAF-mut melanoma cell line 2/59 was engineered for inducible silencing of SEMA6A expression and induction of GFP reporter gene expression. We obtained three polyclonal cell populations: one shCtrl and two shSEMA6A (A3 and H2). Following induction, the expression of SEMA6A was efficiently downregulated in A3 and H2 compared with shCtrl cells (Fig. 2A), resulting in 20-30% reduction in the number of viable melanoma cells (Fig. 2B). In agreement with our previous findings, the depletion of SEMA6A in 2/59 cell model induced actin cytoskeleton remodeling and loss of actin stress fibers (Fig. 2C).

Next, we investigated the involvement of Rho small GTPases and downstream YAP in SEMA6A-induced actin cytoskeletal remodeling. First, we measured the protein levels of Rho GTPs in 2/59 cells, and found the expression of RhoA but not of RhoC small GTPase (data not shown); thus we analyzed the level of active RhoA-GTP upon induction of SEMA6A silencing. As shown in Fig. 2D, SEMA6A depletion caused a reduction of RhoA activity compared with control cells. The densitometric analysis of RhoA-GTP normalized to total RhoA revealed a statistically significant reduction of RhoA activity upon SEMA6A depletion in both H2 and A3 cell populations (Fig. 2E). Next, we found higher levels of YAP phosphorylation following SEMA6A-depletion (Fig. 2A). Moreover, western blot analysis of cytoplasmic and nuclear fractions of control and SEMA6A-depleted cells revealed increased phosphorylation levels of YAP and its cytoplasmic accumulation in A3 and H2 compared with shCtrl cells (Fig. 2F, upper panels). The densitometric analysis of phosphorylated YAP normalized to total YAP confirms increase of YAP phosphorylation in the cytoplasmic fraction of both H2 and A3 SEMA6A-depleted cells (Fig. 2G). YAP cytoplasmic accumulation following SEMA6A depletion was confirmed by immunofluorescence (Fig. 2H and Supplementary S1A, upper panels), and was associated with loss of stress fibers (Fig. 2I, Supplementary S1B, upper panels, and L). We then investigated whether induction of RhoA activity by a specific activator can rescue RhoA-YAP signaling in SEMA6A-depleted cells. To this aim, shCtrl, A3 and H2 cells were treated with RhoA activator and analyzed as above. We observed a significant induction of RhoA activity (Fig. 2D and E) and the reduction of YAP cytoplasmic localization in shCtrl cells compared with their untreated counterpart (Fig. 2F, H, and Supplementary S1A). By contrast, treatment of both A3 and H2 SEMA6A-depleted cell populations with RhoA activator prevented activation of RhoA (Fig. 2D and E) and reduction of YAP cytoplasmic localization (Fig. 2F, H, and Supplementary S1A, lower panels), indicating that SEMA6A is required for downstream activation of RhoA/YAP axis. In agreement, we observed approximately 80% reduction of stress fibers in both A3 and H2 SEMA6A-depleted cells compared with shCtrl cells regardless of stimulation with RhoA activator (Fig. 2I, L, and Supplementary S1B).

These data show that SEMA6A sustains the activity of RhoA that in turn induces YAP nuclear localization. In accordance, SEMA6A depletion is associated with loss of actin stress fibers.

### Dual BRAF/MEK inhibition induces SEMA6A/RhoA/YAP axis

**Fig. 3:**
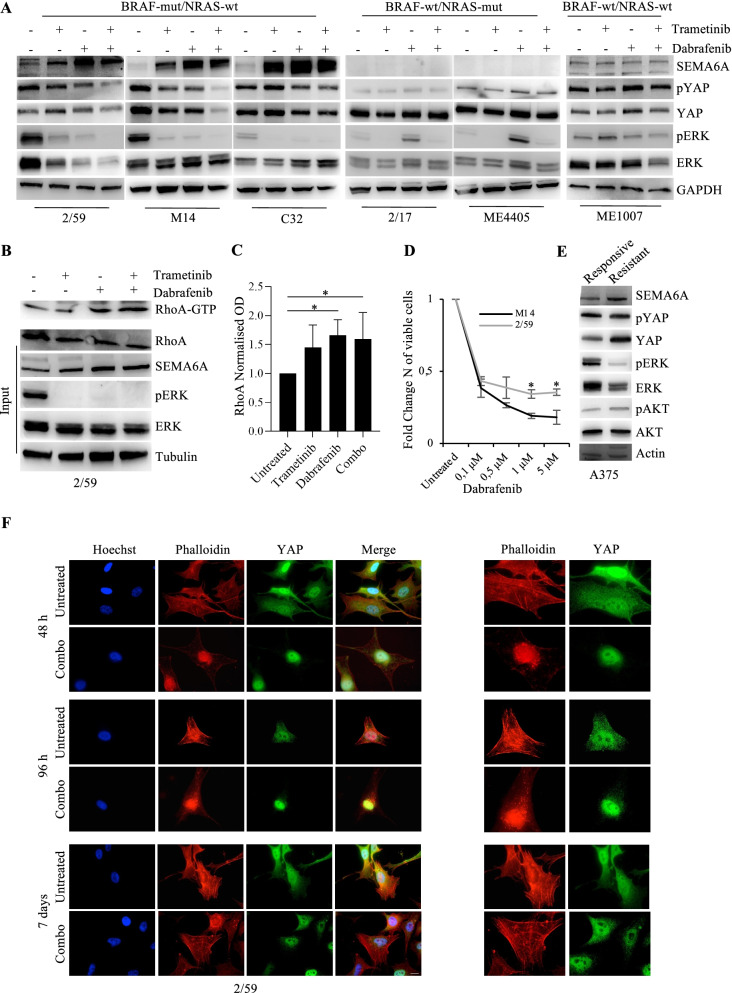
Dabrafenib, trametinib, and their combination induce SEMA6A-RhoA-YAP axis in BRAF-mut melanoma cells. **A**: BRAF-mut/NRAS-wt 2/59, M14 and C32, BRAF-wt/NRAS-mut 2/17 and ME4405, and BRAF-wt/NRAS-wt ME1007 cell lines were treated with 0,1 μM dabrafenib, 5 nM trametinb and their combination for 48 h. Western blot (WB) analysis of SEMA6A, phosphorylated and total YAP and ERK was performed on total cell extracts from untreated and treated cells. The anti-GAPDH antibody was used to validate equivalent amount of loaded proteins in each lane. **B:** WB analysis of activated RhoA (RhoA-GTP) pulled down from cell lysates and RhoA, SEMA6A, phosphorylated and total ERK on total cell extracts from untreated and treated 2/59 cells as specified. The anti-tubulin antibody was used to validate equivalent amount of loaded proteins in each lane. **C:** Densitometric analysis of RhoA-GTP normalized to RhoA is reported. The results are presented as mean+/− standard deviation of three independent experiments (*p < 0.05). **D:** 2/59 and M14 expressing high and low SEMA6A respectively were treated with different doses of dabrafenib for 48 h. The results are reported as fold change of viable cells compared to untreated cells. The results are presented as mean+/− standard deviation of three independent experiments (*p < 0.05). **E:** WB analysis of SEMA6A, phosphorylated and total YAP, ERK, and AKT was performed on total cell extracts from A375 cells sensitive and resistant to dabrafenib. The anti-actin antibody was used to validate equivalent amount of loaded proteins in each lane. **F:** BRAF-mut 2/59 cells plated on poly-l lysine coated slides were treated or not with dabrafenib+trametinib for 48 h, 96 h and 7 days, and stained with anti-YAP (green signal) and Phalloidin (red signal). The cells were counterstained with Hoechst to highlight nuclei. Scale bar 10 μm. Magnification of Phalloidin and YAP images is reported in right most panels

YAP-induced actin cytoskeleton remodeling has been recently reported as a mechanism of resistance to BRAF inhibitors [48, 49]. Thus, we investigated the activity of SEMA6A/RhoA/YAP axis in response to targeted therapy by treating BRAF-mut cell lines 2/59, M14, and C32 with the BRAF inhibitor dabrafenib, the MEK inhibitor trametinib, and the combination of the two drugs. All treatments inhibited the phosphorylation of ERK 1/2 (p-ERK) as expected, and induced a marked increase of SEMA6A expression in all the three BRAF-mut cell lines (Fig. 3A, left panels). The increase of SEMA6A expression was accompanied by a mild but reproducible induction of RhoA activity (Fig. 3B and C) and reduced phosphorylation of YAP (Fig. 3A, left panels). In order to assess whether SEMA6A/RhoA/YAP induction by BRAF and MEK inhibition specifically occurs in a BRAF-mut context, we administered same treatments to 2/17 and ME4405 (BRAF-wt/NRAS-mut), and ME1007 (BRAF-wt/NRAS-wt) cell lines. As expected, p-ERK were inhibited by trametinib and paradoxically induced by dabrafenib, as previously described [51], in NRAS-mut cell lines (Fig. 3A, central panels) and were affected only to a marginal extent in the BRAF-wt/NRAS-wt ME1007 cells (Fig. 3A, right most panel). The treatments did not induce SEMA6A expression, nor the levels of phosphorylated YAP were affected in the BRAF-wt cell lines analyzed (Fig. 3A right panels). These results indicate that BRAF inhibition by dabrafenib, MEK inhibition by trametinib, and dual BRAF/MEK inhibition by dabrafenib+trametinib induce the SEMA6A/RhoA/YAP axis in BRAF-mut but not in BRAF-wt melanoma cells.

Then, we investigated whether SEMA6A expression level is associated with the sensitivity of BRAF-mut cell lines to MAPK inhibition. First, we treated 2/59 and M14 cell lines expressing respectively high (SEMA6A^hi^) and low (SEMA6A^low^) SEMA6A and comparable levels of p-ERK, with different doses of dabrafenib. As shown in Fig. 3D, SEMA6A^hi^ 2/59 cells showed significantly reduced sensitivity to BRAF inhibition compared with SEMA6A^low^ M14. Second, we analyzed by western blot analysis A375 melanoma cell line sensitive to dabrafenib and its dabrafenib-resistant counterpart. We found increased expression of SEMA6A in resistant A375, in association with p-ERK reduction (Fig. 3E). Moreover, YAP expression level was significantly higher in resistant compared with sensitive A375 cells, whereas the two cell lines showed comparable levels of p-YAP, indicating a lower p-YAP/YAP ratio in resistant cells. These data support the involvement of SEMA6A in the mechanisms of resistance of melanoma cells to BRAF inhibition.

**Fig. 4:**
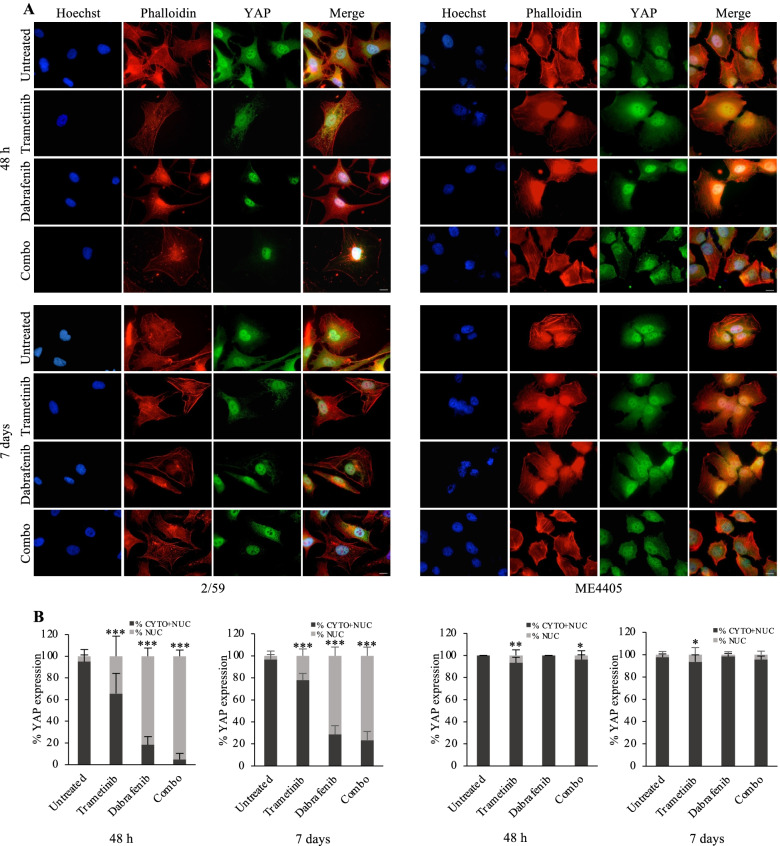
Dabrafenib- and dabrafenib+trametinib-induced SEMA6A-RhoA-YAP axis activation rescues actin cytoskeleton in BRAF-mut melanoma cells. **A:** BRAF-mut 2/59 and BRAF-wt ME4405 cells plated on poly-l lysine coated slides were treated or not with dabrafenib, trametinib and their combination for 48 h and 7 days, and stained with anti-YAP (green signal) and Phalloidin (red signal). The cells were counterstained with Hoechst to highlight nuclei. Scale bar 10 μm. **B:** Quantification of the subcellular localization of YAP from immunofluorescence of 2/59 and ME4405 cells untreated and treated with dabrafenib, trametinib and their combination for 48 h and 7 days. The results are reported as percentage of YAP expression in cytoplasmic + nuclear and nuclear fractions and at least 200 cells were counted per experiment. The results are presented as mean+/− standard deviation of three independent experiments (*p < 0.05; ** *p* < 0,001; *** *p* < 0,0001)

To investigate the dynamics of SEMA6A/RhoA/YAP axis activation and actin cytoskeleton remodeling in response to BRAF and MEK inhibition, we treated BRAF-mut 2/59 and BRAF-wt ME4405 cells up to 7 days and analyzed them by immunofluorescence. Following 48 h dabrafenib+trametinib, BRAF-mut 2/59 cells showed YAP nuclear translocation and loss of actin stress fibers (Fig. 3F, upper panels, and Fig. 4A, left upper panels). Afterwards, surviving cells gradually re-organized their actin cytoskeleton, as shown by images taken 96 h post treatment (Fig. 3F, middle panels), and rescued stress fibers/cytoskeleton organization and YAP cytoplasmic localization after 7 days treatment (Fig. 3F, lower panels and Fig. 4A, left lower panels). Loss of stress fibers and YAP nuclear translocation were mainly induced in 2/59 cells by dabrafenib and dabrafenib+trametinib combination, and only to a lesser extent by trametinib (Fig. 4A, left upper panels). Figure 4B shows the increase in percentage of nuclear YAP upon 48 h and 7 days trametinib (34,4% and 22,2%), dabrafenib (81,6% and 71,3%), and their combination (95,2% and 76,5%) compared with untreated cells (5% and 3,6%). By contrast, in BRAF-wt ME4405 cells, the actin cytoskeleton was not affected by dual BRAF/MEK inhibition (Fig. 4A, right panels), and trametinib only induced a mild YAP nuclear translocation (6,7% at 48 h and 7 days) (Fig. 4A, right panels, and Fig. 4B, right graphs). These data suggest that, in response to dabrafenib and dabrafenib+trametinib, BRAF-mut melanoma cells activate the axis SEMA6A/RhoA/YAP as a compensatory mechanism aimed to sustain actin cytoskeleton remodeling.

Next, we assessed the viability of shCtrl and shSEMA6A cells following drugs treatments. Dabrafenib and dabrafenib+trametinib treatments induced a marked reduction in the number of viable shCtrl cells (Supplementary Fig. S2). As expected, SEMA6A depletion per se reduced the number of viable A3 and more efficiently that of H2 cells compared with shCtrl cells (Supplementary Fig. S2, compare black bars of untreated cells); however, it did not further affect the number of viable cells following drug treatments (Supplementary Fig. S2, compare grey bars of treated cells), suggesting that some other factors may be required for SEMA6A/RhoA/YAP axis to regulate sensitivity to targeted therapies in patients.

### SEMA6A is a mediator of surrounding fibrobalsts-induced melanoma cell growth

**Fig. 5:**
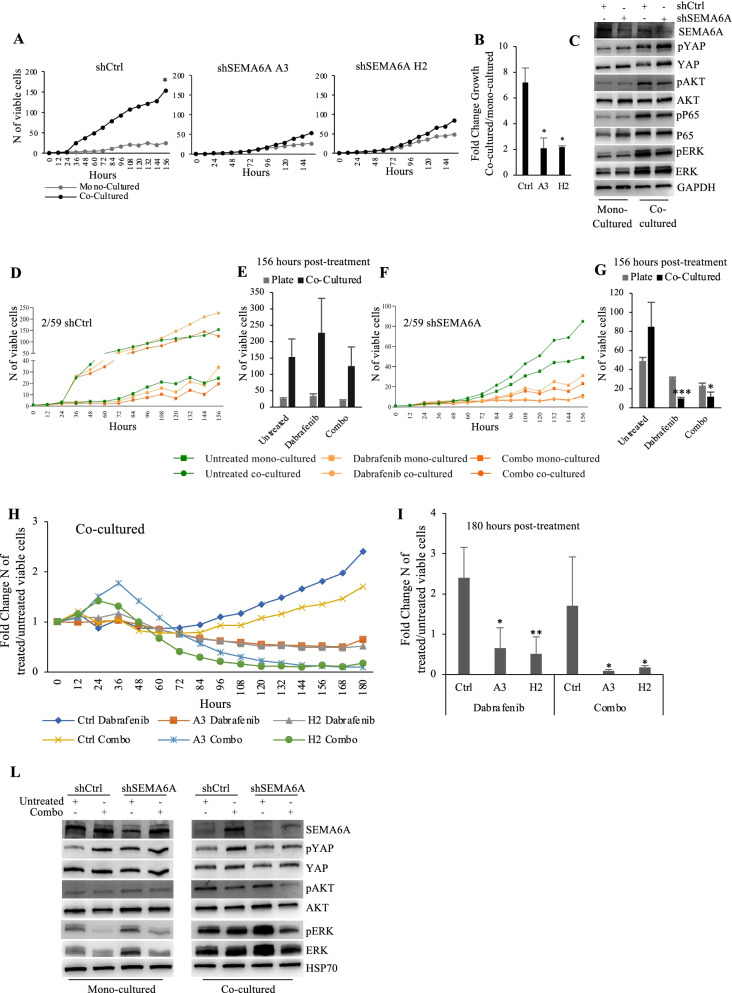
SEMA6A depletion restores responsiveness to dabrafenib and dabrafenib+trametinib in fibroblasts co-cultured BRAF-mut melanoma cells. **A:** Growth curves of mono-cultured and fibroblasts-cocultured shCtrl and SEMA6A-depleted A3 and H2 2/59 cells, periodically monitored up to 156 h. The results are presented as mean+/− standard deviation of three independent experiments (*p < 0.05). **B:** Fold change growth of co-cultured vs mono-cultured shCtrl and SEMA6A-depleted A3 and H2 cells as reported in **A**. **C:** Western blot (WB) analysis of SEMA6A, phosphorylated and total YAP, AKT, P65 and ERK was performed on total cell extracts from shCtrl and SEMA6A-depleted H2 cells cultured in the absence or presence of fibroblasts for 48 h. The anti-GAPDH antibody was used to validate equivalent amount of loaded proteins in each lane. **D** and** F:** Growth curves of mono-cultured and co-cultured shCtrl (**D**) and SEMA6A-depleted H2 cells (**F**), untreated or treated with 0,1 μM dabrafenib and 0,1 μM dabrafenib+ 5 nM trametinib, periodically monitored up to 156 h. **E **and **G:** the number of viable mono-cultured and co-cultured shCtrl (**E**) and SEMA6A-depleted H2 cells (**G**) 156 h post-treatment is reported. The results are presented as mean+/− standard deviation of three independent experiments (*p < 0.05; *** *p* < 0,0001). **H:** Growth curves of fibroblasts-cocultured shCtrl and SEMA6A-depleted A3 and H2 cells, untreated or treated as indicated, periodically monitored up to 180 h. The results are reported as Fold Change number of treated/untreated viable cells. **I:** Fold Change number of treated/untreated viable co-cultured shCtrl and SEMA6A-depleted A3 and H2 cells 180 h post-treatment is reported. The results are presented as mean+/− standard deviation of three independent experiments (*p < 0.05; **p < 0,001). **L:** WB analysis of SEMA6A, phosphorylated and total YAP, AKT, and ERK was performed on total cell extracts from mono-cultured and fibroblasts-cocultured shCtrl and SEMA6A-depleted H2 cells, untreated and treated as indicated for 48 h. The anti-HSP70 antibody was used to validate equivalent amount of loaded proteins in each lane

Sorrounding fibroblasts have been previously reported to promote the growth of melanoma cells in vitro [52]; thus we asked whether microenvironment might play a role in SEMA6A signaling, and exploited a co-culture condition in which melanoma cells are cultured on a monolayer of BJ human fibroblasts. Fibroblasts-cocultured (from hereon, co-cultured) shCtrl had a markedly higher proliferation rate compared with the same cells cultured in the absence of fibroblasts (from hereon, mono-cultured). SEMA6A depletion nearly abolished the proliferative stimulus conferred by fibroblasts (Fig. 5A); indeed, the fibroblasts-dependent fold growth induction was significantly reduced in SEMA6A-depleted cells compared to shCtrl cells (Fig. 5B).

In addition, we sorted GFP-positive shCtrl and SEMA6A-depleted cell populations from co-cultures and analyzed the phosphorylation levels of YAP and of survival and proliferation effectors AKT, P65, and ERK in co-cultured and mono-cultured cells. In agreement with their higher proliferation rate, co-cultured shCtrl cells showed higher phosphorylation levels of YAP, AKT, P65, and ERK compared with their mono-cultured counterpart (Fig. 5C). SEMA6A depletion increased the levels of phosphorylated and total YAP in mono-cultured condition, as expected, and also in co-cocultured cells. Of relevance, SEMA6A depletion reduced the phosphorylation levels of AKT, P65, and ERK only in the presence of sorrounding fibroblasts (Fig. 5C).

These findings recapitulate previous evidence indicating that surrounding fibroblasts sustain the growth of melanoma cells by the activation of pro-survival (PI3K/AKT) and pro-proliferative (MAPK, ΝFκΒ) pathways, and indicate that SEMA6A plays a crucial role as a mediator in this process.

### SEMA6A depletion rescues the efficacy of both BRAF and dual BRAF/MEK inhibition in fibroblasts-cocultured melanoma cells

Tumor microenvironment is known to increase resistance to BRAF inhibitors [32, 33]. Since SEMA6A supports surrounding fibroblasts-induced growth of melanoma cells, we asked whether SEMA6A might affect the efficacy of targeted therapy in co-cultured condition. Thus, we treated mono-cultured and co-cultured shCtrl and SEMA6A-depleted A3 and H2 cell populations with dabrafenib or dabrafenib+trametinib combination, and periodically monitored cell viability over time up to 156 h.

First, we observed reduced efficacy of dabrafenib in co-cultered cells. Indeed, as shown in Fig. 5D, the proliferation rate of both mono-cultured and co-cultured shCtrl cells increased irrespective of treatments; dabrafenib rather conferred a paradoxical growth advantage in co-cultured condition (Fig. 5D and E). Then, we found that in SEMA6A-depleted cells both dabrafenib and dabrafenib+trametinib treatments dramatically reduced cell proliferation only in the co-cultured condition (Fig. 5F). As shown in Fig. 5G, 156 h post-dabrafenib and dabrafenib+trametinib, the number of co-cultured viable SEMA6A-depleted cells was significantly reduced compared with untreated cells. Data from co-cultured shCtrl and SEMA6A-depleted A3 and H2 cells, reported as fold change number of treated/untreated viable cells, show that SEMA6A depletion rescued the efficacy of both dabrafenib and dabrafenib+trametinib (Fig. 5H); indeed, 180 h post-treatment the viability of treated/untreated SEMA6A-depleted cells was significantly reduced as compared with shCtrl cells (Fig. 5I).

Next, we analyzed the levels of phosphorylated and total AKT, ERK, and YAP of both shCtr and shSEMA6A cells mono-cultured and sorted from coculture, following dabrafenib+trametinib treatment. The combination treatment induced SEMA6A expression in mono-cultured shCtrl cells, as expected, and also in co-cultured shCtrl cells. Of relevance, upon dabrafenib+trametinib, SEMA6A depletion reduced the levels of phosphorylated AKT and ERK compared with treated shCtrl cells only in co-cultured condition (Fig. 5L).

Overall these data indicate that surrounding fibroblasts protect shCtrl cells from the inhibitory effect of both BRAF and dual BRAF/MEK inhibition. SEMA6A depletion abrogates this protective effect and rescues the efficacy of both dabrafenib and dabrafenib+trametinib, indicating that SEMA6A plays a key role in mediating fibroblasts-induced insensitivity to targeted therapy.

### SEMA6A predicts progression free survival on dual BRAF/MEK inhibition in BRAF-mut melanoma

**Fig. 6:**
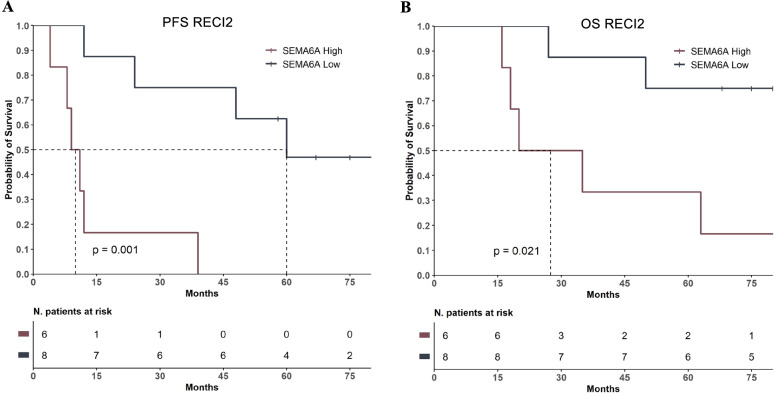
SEMA6A expression predicts low efficacy of dabrafenib+trametinib in BRAF-mut melanoma patients. **A**: Progression-free Survival (PFS) and **B**: Overall Survival (OS) in patients with high and low SEMA6A from the cohort RECI2

Based on the results we obtained in vitro, in the presence of surrounding fibroblasts as a microenvironment-mimicking condition, we investigated the predictive potential of SEMA6A on dual BRAF/MEK inhibition in vivo. To this aim, we analyzed the RECI2 cohort, which includes 14 BRAF-mut advanced melanoma patients admitted to dabrafenib+trametinib therapy at the Regina Elena Cancer Institute. Based on a cut-off value of 12 months of progression-free interval, eight of these patients were considered long-term responders and six short-term responders. Table 3 shows clinicopathological charachteristics of RECI2 cohort. SEMA6A expression was analyzed by IHC and reported both as percentage of positive cells and IS. Patients with high SEMA6A expression had a significantly reduced median PFS and OS compared with those with low SEMA6A (PFS: 10 months vs. 60 months; Log-Rank *p* = 0.001; OS: 27.5 months vs. not reached; Log-Rank *p* = 0.021) (Fig. 6A and B), indicating that SEMA6A might be a good candidate predictor of low efficacy of dual BRAF/MEK inhibition by dabrafenib+trametinib in BRAF-mut melanoma patients.

**Table 3:** Clinicopathological characteristics of RECI2 patients (*N* = 14)

Patient	Gender	Progression-free interval, mo	OS, mo	% of SEMA6A+ cells	SEMA6A IS	Tumor site
**Short-Term Responders**						
1	F	12	35	70%	2	Metastasis
4	M	9	16	60%	2	Lung
10	F	11	18	60%	2	Lymph node
				50%	1	Lung
13	F	4	20	60%	2	Lung and lymph node
14	F	8	98	70%	2	Occipital fragment
15	F	12	27	50%	1	Lung
**Long-Term Responders**						
2	M	Ongoing 67	68	30%	1	Middle meatus
3	F	Ongoing 58	91	30%	1	Lymph node
5	M	39	63	60%	2	Back
6	M	24	50	30%	1	Lung
7	M	60	75	30%	2	Face
8	M	Ongoing 82	83	30%	2	Back
9	M	48	86	30%	2	Lung
11	F	Ongoing 75	80	30%	1	Lymph node

## Discussion

Melanoma is the most lethal form of skin cancer, with BRAF mutations occurring in about 50% of cases. Current targeted therapies include combinations of BRAF and MEK inhibitors and, among the approved ones, dabrafenib+trametinib is associated with a 5-year survival rate of 34% [7]. Following the introduction of CTLA4 and PD1 immune checkpoint inhibitors, the optimal sequence of targeted therapy and immunotherapy for the treatment of patients with BRAF-mut melanoma is under investigation in clinical trials (DREAMseq [NCT02224781] and SECOMBIT [NCT02631447]). In addition, the benefit of combining triplets of immune checkpoint inhibitors and targeted therapy is under evaluation in phase II and III clinical trials [53–55]. With such a growing availability of therapeutic options, predictors of benefit from each class of drugs would be required in order to optimize the choice of treatment and improve patients outcomes.

In the present study, we provide evidence of the involvement of SEMA6A in the biology of BRAF-mut melanoma and the premise for its possible use as a predictive biomarker.

First, we show that SEMA6A protein expression is associated with BRAF mutational status in melanoma patients. SEMA6A has been shown to be deregulated together with many other cancer-related genes in the complex mechanism driven by BRAF^V600E^ on melanoma tumorigenesis [17]; Guo and collegues also indicated the transcription factor MITF as a main mediator of BRAF^V600E^-driven transcription reprogramming. Moreover, a recent RNA-sequencing analysis conducted on SkMel-28 melanoma cells reveals a significant downregulation of SEMA6A following MITF knock out [56], suggesting the possible involvement of MITF in BRAF^V600E^-driven regulation of SEMA6A.

We then unravel the molecular mechanism by which SEMA6A regulates the remodeling of actin cytoskeleton thereby sustaining the aggressive behavior of BRAF-mut melanoma cells, and show the involvement of downstream RhoA-YAP cascade. These findings are in agreement with previous reports showing that the increase in actin filaments promotes YAP nuclear translocation [57, 58]. The functional cross-talk between actin cytoskeleton remodeling and YAP activity offers an explanation of how cells translate SEMA6A-induced cytoskeletal tension into their transcriptional program. Moreover, the identification of a SEMA6A-RhoA-YAP axis adds an important element of knowledge to the upstream regulation of YAP.

Second and to our opinion most relevant, we show that SEMA6A is involved in the mechanisms that regulate the sensitivity of BRAF-mut melanoma cells to dual BRAF/MEK inhibition therapy and acts as a crucial mediator of melanoma-stroma communication.

We show here that both dabrafenib and dabrafenib+trametinib induce the expression of SEMA6A and the downstream RhoA/YAP axis in vitro. SEMA6A induction by BRAF and MEK inhibition has been previously described [59, 60], and higher SEMA6A expression has been reported in BRAF-inhibitor resistant cell lines as compared with sensitive ones [61]. These findings support the role of this semaphorin as crucial player in the mechanisms of resistance to both BRAF and MEK inhibitors. In agreement, we show here that BRAF and dual BRAF/MEK inhibition cause in BRAF-mut melanoma cells an initial disorganization of actin cytoskeleton and the activation of SEMA6A/RhoA/YAP pathway; the resulting massive YAP nuclear translocation functionally determines rescue of actin cytoskeleton remodeling and survival.

However, our results indicate that surrounding fibroblasts play an essential role in coordinating evasion of co-cultured BRAF-mut melanoma cells from both BRAF and combined BRAF/MEK inhibition in a SEMA6A-dependent manner. Indeed, the expression of SEMA6A is associated with insensitivity to both treatments only in co-culturing condition, highlighting the role of SEMA6A as a critical transducer of signals from melanoma-stroma interactions. These data are in agreement with previous findings indicating that upon BRAF inhibition, melanoma-associated fibroblasts provide melanoma cells with drug tolerance [32], and co-culturing of melanoma cells with fibroblasts in vitro conveys protection to the growth inhibitory effects of the BRAF inhibitor vemurafenib [33]. Among the reported mechanisms involved, are increased HGF release by host fibroblasts [30] and the promotion of matrix production and remodeling leading to elevated integrin β1/FAK/SRC signaling in melanoma cells [32]. Moreover, a role of semaphorins in the regulation of microenvironment dynamic has been recently reported [21, 22]. The expression of SEMA3B by cancer cells has been shown to recruit tumor-associated macrophages (TAMs) into the tumor microenvironment by the activation of a neuropilin-mediated signaling pathway that leads to an autocrine release of IL-8 [23], thereby promoting cancer progression. The production of SEMA4D by TAMs in the tumor stroma has been shown to sustain tumor angiogenesis and vessel maturation [24]; while SEMA4D expressed by cancer cells sustains angiogenesis by binding plexin-B1 expressed by endothelial cells [25–27]. Several semaphorins and plexins are expressed by dendritic cells and lymphocytes [28, 29], suggesting a role of these proteins in the immune response. Overall these evidences foster the role of semaphorins in instructing stroma cells to perform specific functions, mainly aimed at cancer progression. However there is no previous evidence of the role of SEMA6A in melanoma-microenvironment interactions.

Our in vitro results were strongly supported by data obtained from a cohort of BRAF-mut melanoma patients admitted to dabrafenib+trametinib treatment. Indeed, we showed that SEMA6A expression is associated with short-time benefit from dual BRAF/MEK inhibition. Despite a small number of patients was included in this cohort, they were homogeneously distributed between short-term and long-term responders, and the association between SEMA6A expression and recurrence-free interval was highly significant and high SEMA6A expression was detected in all the short-term responders. Unfortunately, we could not extend our analysis in publicly available larger cohorts, due to unavailability of mRNA data from BRAF-mut patients admitted to dual BRAF/MEK targeted therapy. Thus, further investigation in larger patient cohorts will be required in order to assess the use of SEMA6A as predictive biomarker for dual BRAF/MEK targeted therapy.

## Conclusion

Overall, our study indicates that SEMA6A is a crucial player in the biology of BRAF-mut melanoma, with high protein expression being associated with worse prognosis in term of OS and PFS of advanced disease and shorter relapse-free interval of early disease. Mechanistically, SEMA6A activates a RhoA/YAP axis that functionally results in actin cytoskeleton remodeling. Furthermore, dual BRAF/MEK inhibition induces early actin cytoskeleton disruption and activates SEMA6A/RhoA/YAP signaling that finally results in rescue of actin cytoskeleton and reduced efficacy of targeted therapy in a microenvironment-mimicking culture condition. Finally, SEMA6A protein expression predicts shorter recurrence-free interval in patients treated with dual BRAF/MEK inhibition. Thus, the possible use of SEMA6A protein assessment as predictive biomarker in BRAF-mut melanoma deserves further investigation.

## Supplementary Information

**Additional file 1: Supplementary Fig. S1.** A: shCtr and SEMA6A-depleted A3 cells plated on poly-l lysine coated slides were treated or not with RhoA activator and stained with anti-YAP (red signal) or B: with Phalloidin (red signal). GFP reporter gene expression revealed successful silencing induction. The cells were counterstained with Hoechst to highlight nuclei. Scale bar 10 μm. **Supplementary Fig. S2.** Fold change number of viable shCtrl and SEMA6A-depleted A3 and H2 cells untreated and treated for 48 h with 0,1 μM dabrafenib (A) and 0,1 μM dabrafenib+ 5 nM trametinib (B) as compared with untreated shCtrl 2/59 cells. The results are presented as mean +/− standard deviation of three independent experiments.

## Data Availability

The datasets used and/or analyzed during the current study are available from the corresponding author on reasonable request.

## Abbreviations

SEMA6A: Semaphorin 6A
RhoA: Ras homolog family member A
YAP: Yes-associated protein
BRAF: BRAF proto-oncogene, serine/threonine kinase
NRAS: NRAS proto-oncogene, GTPAase
MEK: Mitogen-activated protein kinase kinase
MAPK: Mitogen-activated protein kinase
RECI: Regina Elena Cancer Institute
TCGA: The cancer genome atlas
DFCI: Dana-Farber Cancer Institute
OS: Overall survival
PFS: Progression-free survival
RFS: Relapse-free survival
CAFs: Cancer-associated fibroblasts
IHC: Immunohistochemistry
IS: Intensity score
GFP: Green fluorescent protein
MITF: Melanocyte inducing transcription factor
TAMs: Tumor-associated macrophages
WB: Western blot
