# Supplementary material for: SEMA6A/RhoA/YAP axis mediates tumor-stroma interactions and prevents response to dual BRAF/MEK inhibition in BRAF-mutant melanoma
Source: J Exp Clin Cancer Res. 2022 Apr 19;41:148. doi: 10.1186/s13046-022-02354-w (PMC9016967; doi:10.1186/s13046-022-02354-w)
Supplement: Supplementary file 1 — Additional file 1: Supplementary Fig. S1. A: shCtr and SEMA6A-depleted A3 cells plated on poly-l lysine coated slides were treated or not with RhoA activator and stained with anti-YAP (red signal) or B: with Phalloidin (red signal). GFP reporter gene expression revealed successful silencing induction. The cells were counterstained with Hoechst to highlight nuclei. Scale bar 10 μm. Supplementary Fig. S2. Fold change number of viable shCtrl and SEMA6A-depleted A3 and H2 cells untreated and treated for 48 h with 0,1 μM dabrafenib (A) and 0,1 μM dabrafenib+ 5 nM trametinib (B) as compared with untreated shCtrl 2/59 cells. The results are presented as mean +/− standard deviation of three independent experiments. [file 13046_2022_2354_MOESM1_ESM.pdf]

Supplementary Figure S1

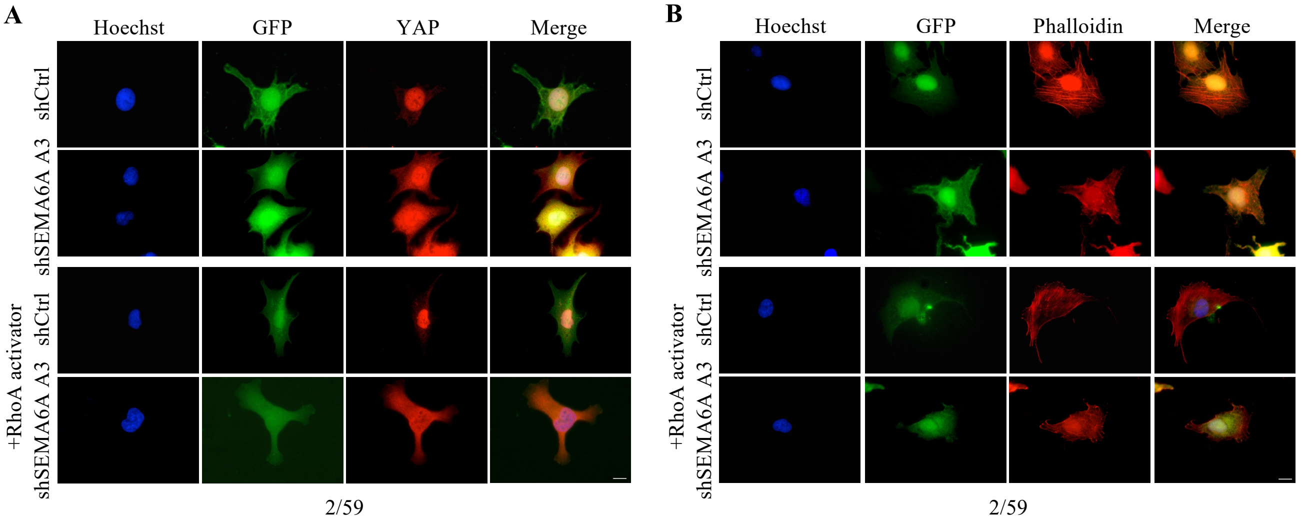

A: shCtr and SEMA6A-depleted A3 cells plated on poly-l lysine coated slides were treated or not with RhoA activator and stained with anti-YAP (red signal) or **B**: with Phalloidin (red signal). GFP reporter gene expression revealed successful silencing induction. The cells were counterstained with Hoechst to highlight nuclei. Scale bar 10 µm.

Supplementary Figure S2

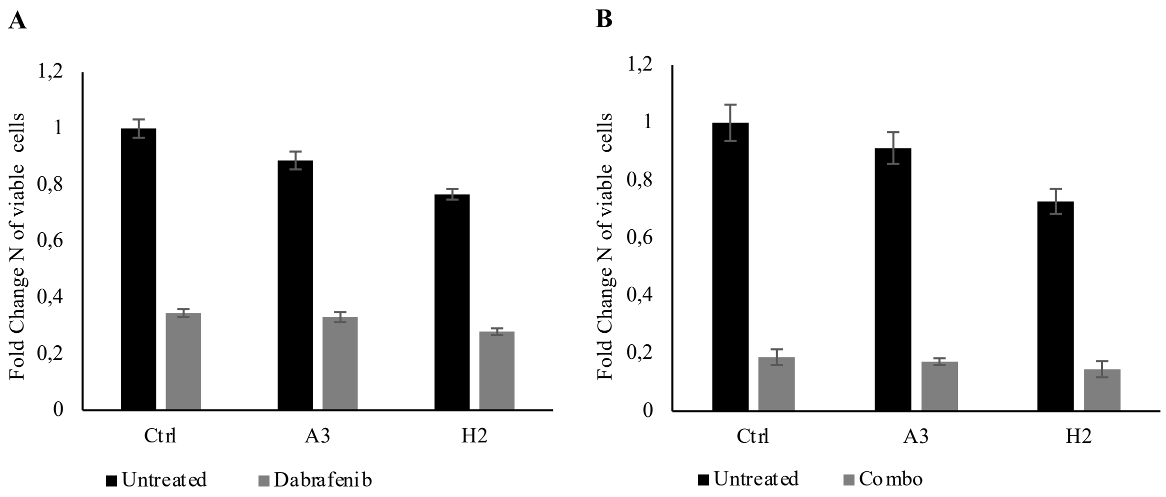

Fold change number of viable shCtrl and SEMA6A-depleted A3 and H2 cells untreated and treated for 48 hours with 0,1 µM dabrafenib (A) and 0,1 µM dabrafenib+5nM trametinib (B) as compared with untreated shCtrl 2/59 cells. The results are presented as mean +/- standard deviation of three independent experiments.
